# TAVR-in-TAVR with a balloon-expandable valve for paravalvular leak

**DOI:** 10.3389/fcvm.2024.1374078

**Published:** 2024-03-19

**Authors:** Takashi Nagasaka, Vivek Patel, Ofir Koren, Alon Shechter, Tarun Chakravarty, Wen Cheng, Hideki Ishii, Hasan Jilaihawi, Mamoo Nakamura, Raj R. Makkar

**Affiliations:** ^1^ Cedars-Sinai Medical Center, Smidt Heart Institute, Los Angeles, CA, United States; ^2^ Department of Cardiovascular Medicine, Gunma University Graduate School of Medicine, Maebashi, Japan; ^3^ Bruce Rappaport Faculty of Medicine, Technion Israel Institute of Technology, Haifa, Israel; ^4^ Sackler School of Medicine, Tel Aviv University, Tel Aviv, Israel

**Keywords:** paravalvular leak, transcatheter aortic valve replacement, TAVR-in-TAVR, New York heart association functional class, balloon-expandable valve

## Abstract

**Introduction:**

Paravalvular leak (PVL) is a severe complication of transcatheter aortic valve replacement (TAVR) that can lead to poor outcomes. TAVR-in-TAVR is a promising treatment for PVL; however, reports on its safety or efficacy are limited. In this study, we aimed to investigate the clinical outcomes of TAVR-in-TAVR using balloon-expandable prostheses for PVLs after TAVR.

**Methods:**

We retrospectively analyzed data from patients who underwent TAVR-in-TAVR using balloon-expandable Sapien prostheses for PVL after an initial TAVR at our institution. The procedural success, in-hospital complications, all-cause mortality, and echocardiographic data for up to 2 years post-surgery were evaluated.

**Results:**

In total, 31 patients with a mean age of 81.1 ± 7.9 years and mean Society of Thoracic Surgeons score of 8.8 ± 5.4% were identified. The procedural success rate of TAVR-in-TAVR was 96.8% (30/31). No in-hospital deaths, cardiac tamponade, or conversion to sternotomy occurred. Re-intervention was performed in only one patient (3.2%) during hospitalization. The all-cause mortality rates at 30 days and 2 years were 0% and 16.1%, respectively. A significant reduction in the PVL rate was observed at 30 days compared with that at baseline (*p* < 0.01).

**Discussion:**

Our findings suggest that TAVR-in-TAVR using balloon-expandable prostheses is safe and effective for PVL after TAVR with low complication rates and acceptable long-term outcomes. Further studies with larger sample sizes are needed to confirm our findings.

## 1 Introduction

Transcatheter aortic valve replacement (TAVR) is a standard treatment for patients with intermediate or high risks of severe aortic stenosis (AS) (1–5). Following the PARTNER 3 trial, TAVR was established as an alternative treatment for younger patients and those with a low risk of aortic stenosis (6). Moreover, new-generation prosthetic valves have contributed to the reduction of procedural complications and improvement in clinical outcomes (7–9), such as reduced rates of paravalvular leak (PVL) (10–12). Nonetheless, PVL remains a notable complication and is observed more frequently in patients undergoing TAVR than in those undergoing surgical aortic valve replacement (SAVR) (13, 14). This increased incidence of PVL after TAVR can be attributed to the difficulty in removing the calcified native valve and directly visualizing the annulus. The indications of TAVR continue to increase, and the incidence of PVL is expected to increase. PVL after TAVR can increase the risk of mortality (10, 15–17), and several studies have shown that mild PVL can also result in unfavorable clinical outcomes (18, 19). Hence, prompt detection and management of PVL after TAVR are paramount for optimizing patient outcomes.

While conventional surgical treatment is generally effective in treating PVL (20), the majority of patients have a high risk or are inoperative for sternotomy after TAVR. Percutaneous PVL closure is minimally invasive and effective in reducing the incidence of PVL (21–23). However, it may not be a viable option when PVL is caused by an undersized prosthetic valve or improper positioning during TAVR. Under such circumstances, TAVR-in-TAVR is considered an alternative approach to preventing PVL, and several case reports have demonstrated its feasibility and success (24–26). In recent years, TAVR-in-TAVR has become popular for treating structural valve degeneration after TAVR or SAVR. Some studies have reported that TAVR-in-TAVR for structural valve degeneration is associated with favorable outcomes, including reduced rates of mortality and complications and improvement in symptoms (27–29). However, few studies have attempted to systematically investigate the clinical outcomes of TAVR-in-TAVR in patients with PVL. This necessitates the assessment of the safety and efficacy of TAVR-in-TAVR for PVL.

Therefore, this study aimed to evaluate the procedural and clinical outcomes of patients who underwent TAVR-in-TAVR for PVL.

## 2 Methods

### 2.1 Patient population and procedures

This retrospective study included consecutive patients who underwent TAVR-in-TAVR for PVL at Cedars Sinai Medical Center between April 2016 and March 2021. The decision to perform re-intervention for PVL was clinically driven. All patients with PVL after TAVR at our institution were evaluated, and their eligibility for TAVR-in-TAVR was discussed by a multidisciplinary cardiac team. The procedural approach and specifications, including valve type, valve size, pre- and post-implantation balloon dilatation, supplementary use of vascular plugs, and medication after the procedures, were determined by the surgeon based on the preoperative cardiac computed tomography (CT) and echocardiography findings. The study adhered to the principles of the Declaration of Helsinki (1975), informed consent was obtained from all patients, and the protocol was approved by the Institutional Review Board of the institution. Patient information (demographics; laboratory measurements; medical history; procedure data, including procedural outcomes; and follow-up data) was retrospectively obtained from an established interventional cardiology laboratory database at our institution, records of outpatient visits, and telephone interviews. All procedures were performed through the femoral arteries under fluoroscopic and echocardiographic guidance and general anesthesia. Balloon-expandable Edwards Sapien 3 and Sapien Ultra prostheses (Edwards Lifesciences, Irvine, California, USA) were used as second implantation valves for all TAVR-in-TAVR procedures.

### 2.2 Echocardiography assessment

Transthoracic echocardiography was performed before the procedure and PVL was assessed and classified as none, trace, mild, moderate, or severe, based on the recommended echocardiography criteria (30). Follow-up transthoracic echocardiography was performed at 30 days, 1 year, and 2 years after TAVR. Prosthesis-patient mismatch (PPM), which refers to the effective orifice area indexed to the body surface area of the bioprosthetic valve, was classified as severe for values ≤0.65 cm^2^/m^2^, moderate for values between 0.65 and 0.85 cm^2^/m^2^, or none for values >0.85 cm^2^/m^2^, according to the Valve Academic Research Consortium 2 criteria (31, 32). The assessment of PVL origins, either from insufficient apposition or undersized prostheses, utilized echocardiography and CT imaging. We considered PVL to be due to inadequate apposition when the TAVR valve had not reached its optimal, designed diameter or when eccentricity was observed. Conversely, if PVL persisted despite the valve being expanded to its nominal diameter, it was attributed to the prosthesis being too small.

### 2.3 CT image evaluation

ECG-gated contrast-enhanced multi-detector CT (MDCT) examinations were conducted using a 3-dimensional CT workstation (3mensio, Pie Medical Imaging, Bilthoven, Netherlands) before the TAVR-in-TAVR by experts in a dedicated CT core laboratory at our institution. We evaluated the size of the transcatheter heart valve (THV) on the annulus and the coronary height from the THV of the first TAVR using cardiac CT. The virtual THV-to-coronary artery (VTC) distance was also measured to assess the risk of coronary artery occlusion; the VTC distance is defined as the distance between the simulated THV and the ostium of coronary arteries on the short-axis image (33).

### 2.4 Definitions of procedural success and clinical outcomes

Procedural success was defined as follows: proper positioning of the device with at least one grade reduction of PVL to mild or less on intraoperative echocardiography; successful access, delivery, and retrieval of the device system; and no surgical conversion related to the procedure. In-hospital complications, including in-hospital death, cardiac tamponade, stroke, acute coronary obstruction, new permanent pacemaker implantation, new atrial fibrillation, major bleeding, major vascular complications, acute kidney injury, and aortic valve re-intervention, were investigated according to the Valve Academic Research Consortium 2 recommendations (34). The clinical outcomes of interest were all-cause mortality and rehospitalization due to heart failure (HF) for up to 2 years following the procedure. Additionally, we measured changes in the echocardiographic parameters, laboratory parameters, and New York Heart Association (NYHA) class after treatment.

### 2.5 Statistical analysis

Continuous variables are expressed as mean ± standard deviation or median and interquartile range (IQR). Categorical variables are expressed as numbers and percentages. Differences in the characteristics and events between the follow-up periods were evaluated using the unpaired Student's *t*-test or Mann–Whitney test for continuous variables. The chi-squared or two-tailed Fisher's exact test was used to compare discrete variables, and the Wilcoxon signed-rank test was used to compare paired ordinal variables. The cumulative event rates were estimated using Kaplan–Meier survival analysis. *P*-values < 0.05 denoted statistical significance. All analyses were conducted using SPSS software version 24.0 (IBM Corp., Armonk, NY, USA).

## 3 Results

Thirty-one patients underwent TAVR-in-TAVR for PVL after their first TAVR. The baseline characteristics of the patients are summarized in Table 1. The mean patient age was 81.1 ± 7.9 years; 24 (77.4%) were male. The overall Society of Thoracic Surgery score for mortality was 8.8 ± 5.4%. Echocardiographic data showed that the mean left ventricular ejection fraction and transvalvular gradient were 53.1 ± 14.4% and 15.7 ± 11.0 mmHg, respectively (Table 2). A PVL grade of moderate or worse at baseline was observed in 87.1% of the patients (54.8% moderate, 32.3% severe). The mean area and perimeter of THV before TAVR determined using CT were 446.5 ± 126.9 mm^2^ and 74.2 ± 10.5 mm, respectively. The mean VTC distance for the left and right coronary arteries were 5.5 ± 2.2 mm and 4.4 ± 1.7 mm, respectively.

**Table 1:** Baseline characteristics of the patients (*N* = 31).

Characteristic	*n* (%)
Age, years	81.1 ± 7.9
Male	24 (77.4)
BSA	1.94 ± 0.2
Hypertension	29 (93.5)
Dyslipidemia	22 (71.0)
Diabetes	8 (25.8)
Smoker	15 (48.4)
Previous CVA/TIA	8 (25.8)
Porcelain aorta	2 (6.5)
Chronic lung disease	8 (25.8)
Coronary artery disease	17 (54.8)
Peripheral artery disease	6 (19.4)
Previous myocardial infarction	3 (9.7)
Previous percutaneous coronary intervention	6 (19.4)
Previous coronary artery bypass graft	5 (16.1)
Atrial fibrillation	13 (41.9)
Previous permanent pacemaker implantation	6 (20.0)
Chronic kidney disease^a^	13 (41.9)
Current dialysis	1 (3.2)
Bicuspid	3 (9.7)
STS score, %	8.8 ± 5.4
Surgical risk group by STS score	
Low risk	7 (22.6)
Intermediate risk	9 (29.0)
High risk	15 (48.4)
NYHA functional class	
I	0 (0)
II	1 (3.2)
III	16 (51.6)
IV	14 (45.2)
Laboratory analysis	
Creatinine, mg/dl	1.39 ± 0.84
B-type natriuretic peptide, pg/mL	834.4 ± 957.9
Medications	
Aspirin	22 (71.0)
P2Y12 inhibitor	8 (25.8)
Anticoagulation	14 (45.2)
Beta-blocker	25 (80.1)
ACE/ARB	21 (67.7)
Statin	27 (87.1)

Values are presented as *n* (%) or mean ± SD.

^a^ Estimated glomerular filtration rate <60 ml/min/1.73 m^2.^

BSA, body surface area; CVA/TIA, cerebrovascular accident/transient ischemic attack; STS, society of thoracic surgery; NYHA, New York heart association; ACE/ARB, ACE-I, angiotensin-converting enzyme inhibitors/ angiotensin-receptor blockers.

**Table 2:** Echocardiographic characteristics and computed tomography findings at baseline.

Echocardiography finding	
Left ventricular ejection fraction, %	53.1 ± 14.4
PVL grade after first TAVR	
2	4 (12.9)
3	17 (54.8)
4	10 (32.3)
Effective orifice area, cm^2^	1.49 ± 0.51
Mean aortic valve gradient, mm Hg	15.7 ± 11.0
Pulmonary arterial systolic pressure, mm Hg	39.4 ± 18.9
Mitral regurgitation ≥ moderate	11 (35.5)
Tricuspid regurgitation ≥ moderate	7 (22.6)
Computed tomography	
THV area, mm^2^	446.5 ± 126.9
Perimeter of THV, mm	74.2 ± 10.5
LCA height to the THV plane, mm	15.1 ± 5.8
RCA height to the THV plane, mm	17.5 ± 4.2
LCA distance from THV, mm	5.5 ± 2.2
RCA distance from THV, mm	4.4 ± 1.7

Values are presented as *n* (%) or mean ± SD.

PVL, paravalvular leak; TAVR, transcatheter aortic valve replacement; THV, transcatheter heart valve; RCA, right coronary artery; LCA, left coronary artery.

### 3.1 Procedural data

The details of the individual TAVR-in-TAVR procedures are presented in Table 3. The duration between the TAVR-in-TAVR for PVL and the first TAVR was 404 days (IQR: 163–726 days). Sapien 3 and Sapien Ultra were used in 21 (67.7%) and 10 (32.3%) patients, respectively. Concomitant procedures were performed as follows: percutaneous PVL closure with vascular plugs in three (9.7%), pre-implantation balloon dilatation in four (12.9%), post-implantation balloon dilatation in eight (25.8%), and percutaneous coronary intervention in one (3.2%) patient. None of the patients underwent the chimney, bioprosthetic, or native aortic scallop intentional laceration to prevent iatrogenic coronary artery obstruction. The average fluoroscopy duration and contrast volume for the procedure were 15.7 ± 9.8 min and 64.6 ± 53.5 ml, respectively. The median length of hospital stay was 2 days (IQR: 1–4 days). The procedural details of the first TAVR are presented in Supplementary Table S1.

**Table 3:** Procedural data for TAVR-in-TAVR.

Patient	Time from the first TAVR, days	Cause of PVL	Access site	Urgent	Redo TAVR Device (Sapien)	THV size, mm	Cerebral protection system	Vascular plug use	Pre-dilatation balloon	Post-dilatation balloon	Fluoroscopy time, min	Contrast volume, mL	Length of hospital stay, days
1	243	Undersized	FA	No	Ultra	26	Yes	No	Yes	No	12	40	3
2	42	Undersized	FA	No	3	29	No	No	No	No	15	10	3
3	852	Low implant	FA	No	3	23	No	No	No	No	18	25	4
4	7	Undersized	FA	No	Ultra	29	No	No	Yes	No	12	3	9
5	513	High implant	FA	No	Ultra	26	No	No	No	No	16	300	1
6	191	Low implant	FA	No	3	29	No	No	No	No	15.1	40	1
7	521	Low implant	FA	Yes	Ultra	23	No	No	No	No	12	40	4
8	601	Undersized	FA	No	3	23	No	No	No	No	9	10	2
9	853	Undersized	FA	No	3	29	No	No	No	No	12	60	7
10	304	Incomplete valve adherence	FA	No	3	26	No	Yes	No	No	19.9	50	2
11	70	Undersized	FA	No	3	23	No	No	No	No	25	100	3
12	0	Undersized	FA	No	3	29	No	No	No	Yes	63.1	120	1
13	585	High implant	FA	No	3	26	No	Yes	No	No	9.3	50	1
14	1,009	Undersized	FA	Yes	3	23	No	No	No	No	14.8	90	4
15	142	Low implant	SCA	No	3	26	No	No	No	Yes	18.6	100	4
16	404	Unknown	FA	No	3	29	No	No	No	No	6.3	50	4
17	1,036	Low implant	FA	No	Ultra	29	No	No	No	Yes	16	90	1
18	1,927	Undersized	FA	No	3	29	No	No	No	No	15.2	80	1
19	293	Low implant	FA	Yes	3	26	No	No	No	No	9.3	70	2
20	49	Incomplete valve adherence	FA	No	Ultra	26	Yes	No	No	No	17	60	1
21	1,366	Unknown	SCA	No	Ultra	26	No	No	No	Yes	15	70	3
22	298	Undersized	FA	Yes	3	29	No	No	No	No	13	70	5
23	404	Undersized	FA	No	3	26	Yes	No	Yes	No	15	30	1
24	211	High implant	FA	No	3	26	Yes	No	No	Yes	14.9	60	1
25	1,551	Incomplete valve adherence	FA	No	Ultra	26	No	No	No	Yes	23	120	1
26	466	Undersized	FA	No	Ultra	26	Yes	No	No	No	12.5	30	1
27	508	Undersized	FA	No	3	26	No	No	No	No	9	40	1
28	66	Low implant	FA	No	3	29	No	No	No	No	7	30	1
29	1,193	Incomplete valve adherence	FA	No	3	26	No	Yes	No	Yes	10.2	70	1
30	86	High implant	FA	Yes	Ultra	29	No	No	No	Yes	13	70	5
31	184	Undersized	FA	No	3	23	No	No	Yes	No	18	25	4

PVL, paravalvular leak; TAVR, transcatheter aortic valve replacement; THV, transcatheter heart valve; FA, femoral artery; SCA, subclavian artery.

The outcomes of the TAVR-in-TAVR group are outlined in Table 4. Procedural success was achieved in 30 (96.8%) patients. There was no in-hospital death, cardiac tamponade, acute coronary obstruction, or conversion to sternotomy. Transient ischemic attack or non-disabling stroke occurred in two patients (6.5%); however, there was no case of disabling stroke. However, one patient required re-intervention for severe PVL during hospitalization following TAVR-in-TAVR.

**Table 4:** In-hospital outcomes.

Outcome	*n* (%)
Procedural success	30 (96.8)
In-hospital death	0 (0)
Cardiac tamponade	0 (0)
Transient ischemic attack/non-disabling stroke	2 (6.5)
Disabling Stroke	0 (0)
Acute coronary obstruction	0 (0)
New permanent pacemaker implantation	1 (3.2)
New atrial fibrillation	0 (0)
Major or life-threatening bleeding	1 (3.2)
Major vascular complication	1 (3.2)
Acute kidney injury	1 (3.2)
Re-intervention	1 (3.2)
Conversion to sternotomy	0 (0)

Values are presented as *n* (%).

### 3.2 Clinical outcomes

**Figure 1:**
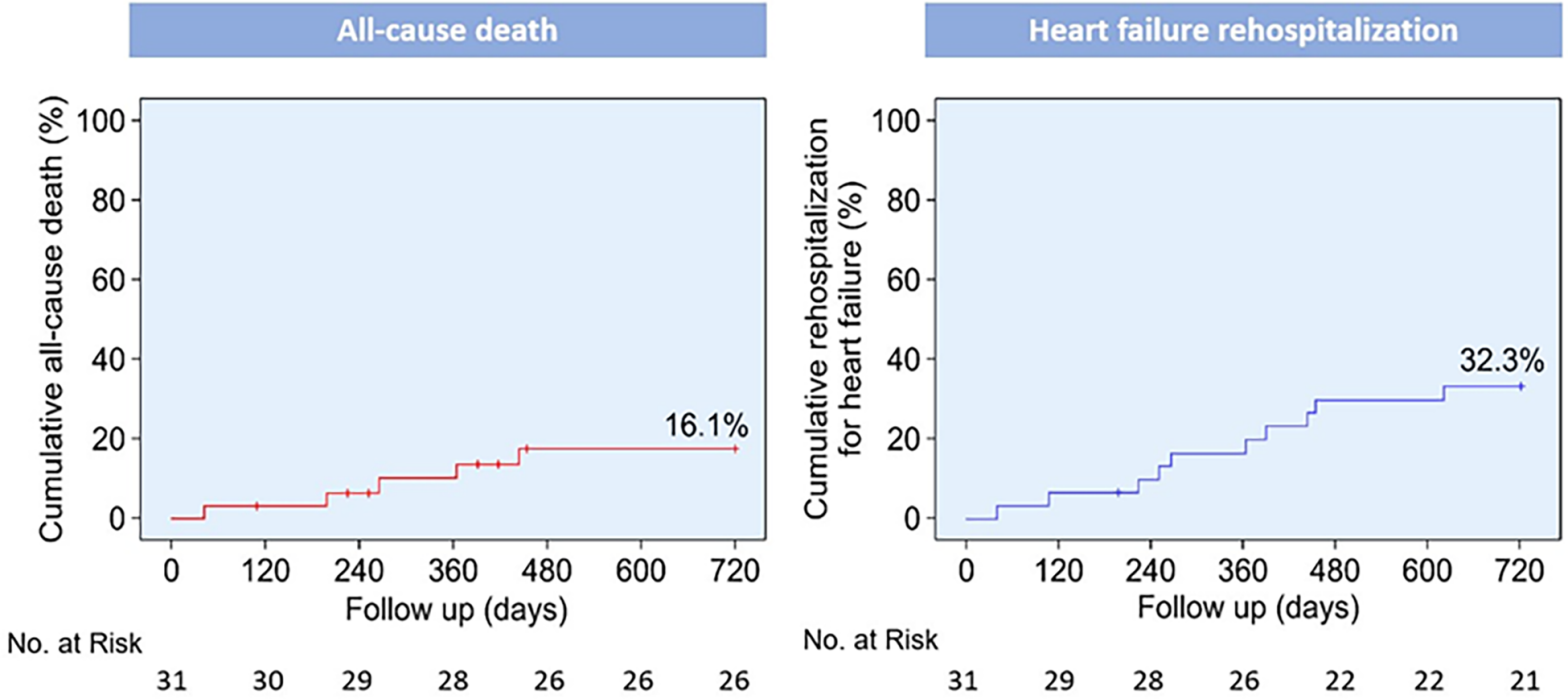
Kaplan–Meier curves for clinical outcomes up to 2 years post-procedure. Kaplan–Meier curves for all-cause death and rehospitalization for heart failure.

The median follow-up duration was 586 days (IQR: 417–740 days), and no patients were lost to follow-up. The rates of all-cause mortality and HF rehospitalization were 0 (0%) and 5 (16.1%) at 30 days (Table 5) and 1 (3.2%) and 10 (32.3%) at 2 years, respectively. At the 2-year follow-up, four patients succumbed to cardiovascular disease, whereas one patient succumbed to sepsis. Additionally, three patients (9.7%) had experienced non-disabling or disabling stroke; one (3.2%) had experienced myocardial infarction, and two (6.5%) had experienced major or life-threatening bleeding. The 2-year Kaplan–Meier curves for the incidence of all-cause mortality and HF-related rehospitalization are depicted in Figure 1.

**Table 5:** Clinical outcomes after repeat TAVR.

30-day outcomes	*n* (%)
All-cause mortality	0 (0)
Readmission for heart failure	1 (3.2)
Cardiovascular death	0 (0)
Non-disabling or disabling stroke	2 (6.5)
Myocardial infarction	0 (0)
Major or life-threatening bleeding	1 (3.2)
2-year outcomes	*n* (%)
All-cause mortality	5 (16.1)
Readmission for heart failure	10 (32.3)
Cardiovascular death	4 (12.9)
Non-disabling or disabling stroke	3 (9.7)
Myocardial infarction	1 (3.2)
Major or life-threatening bleeding	2 (6.5)

Values are presented as *n* (%). TAVR, transcatheter aortic valve replacement.

### 3.3 Functional status

**Figure 2:**
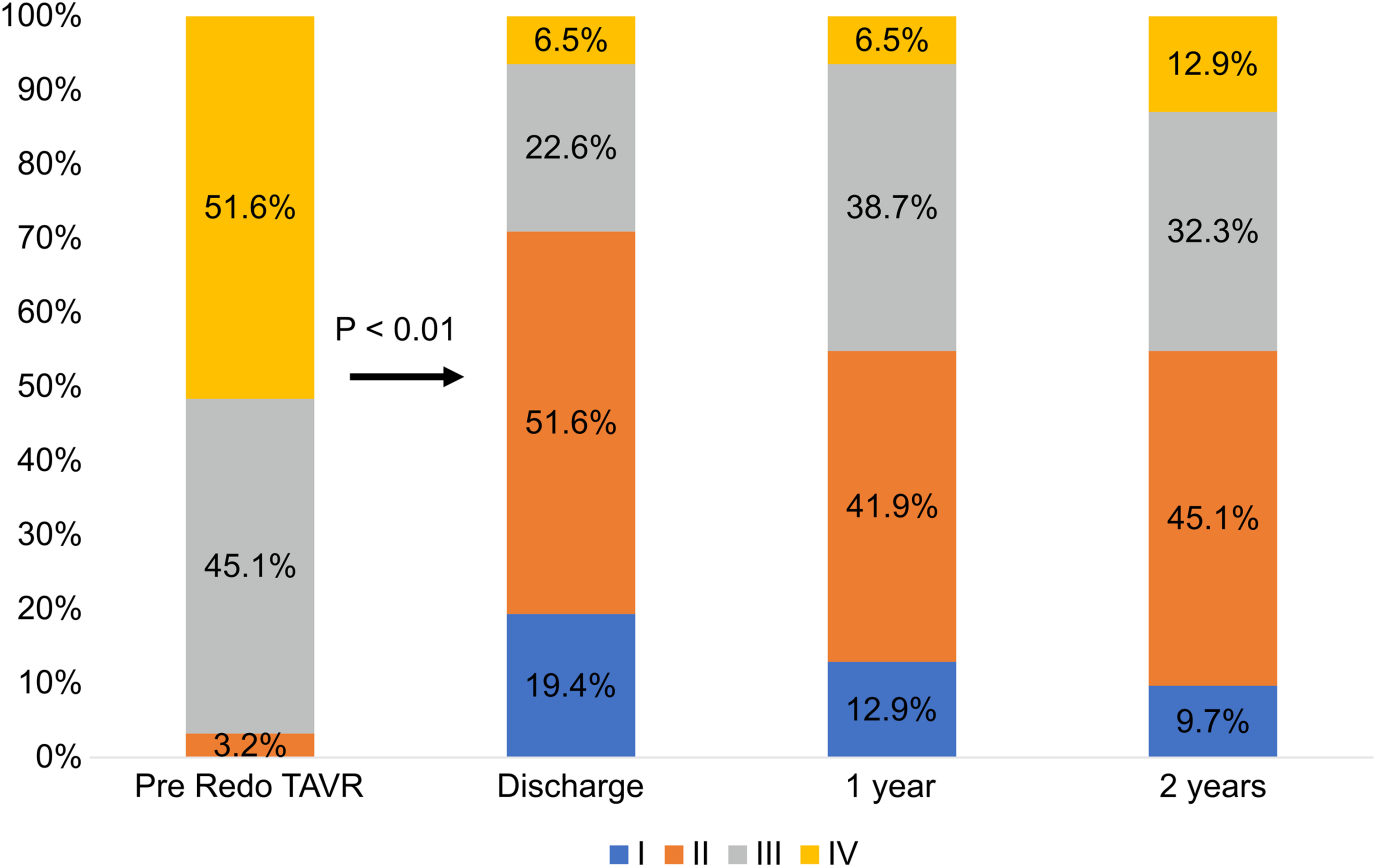
Changes in the New York heart association (NYHA) functional class. TAVR, transcatheter aortic valve replacement.

Figure 2 illustrates the changes in the NYHA class evaluated as a measure of functional capacity in patients at follow-up. Twenty-six (83.9%) patients had improved by at least one class from baseline at 30 days. At 2 years, 22 patients (70.9%) demonstrated significant improvement from their baseline (*p* < 0.01).

### 3.4 Echocardiographic outcomes and laboratory parameters

**Figure 3:**
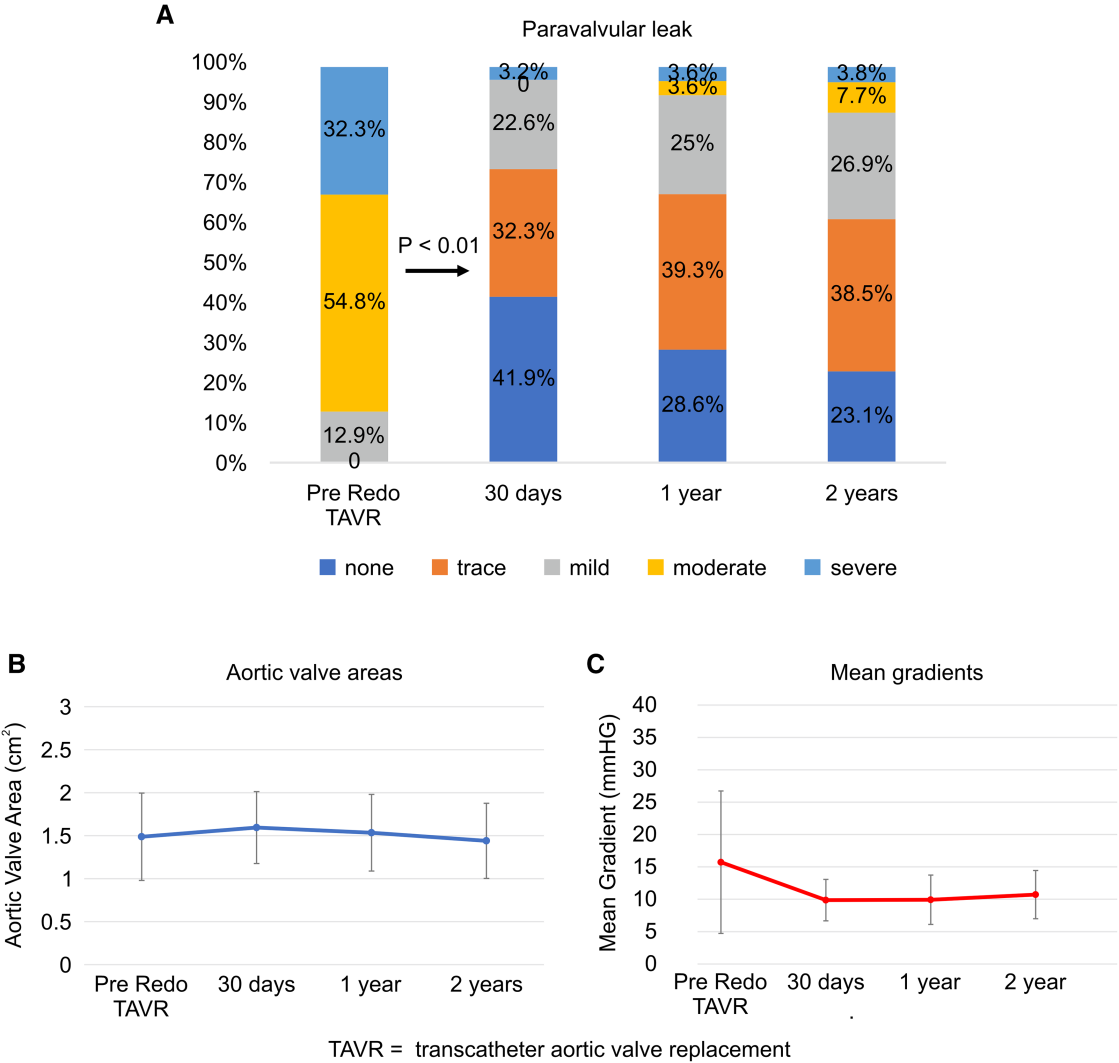
Paravalvular leak, aortic valve area, and mean gradient at follow-up. (**A**) Paravalvular leak, (**B**) Aortic valve areas, (**C**) Mean gradients. TAVR, transcatheter aortic valve replacement.

**Figure 4:**
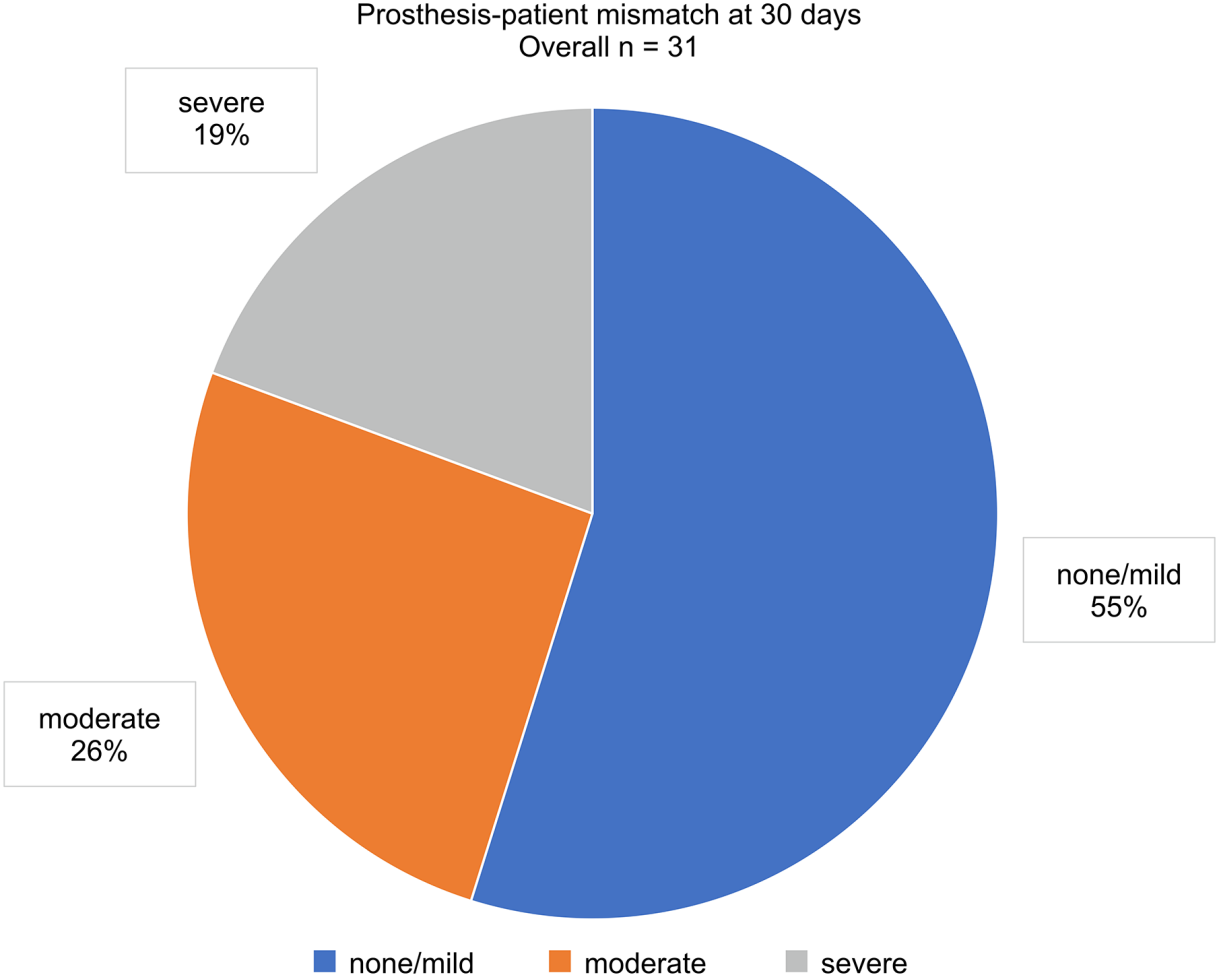
Prosthesis–patient mismatch at 30 days.

The PVL grade at discharge significantly improved from that at baseline (*p* < 0.01; Figure 3A). Over 2 years, 88.5% of the patients maintained a PVL grade mild or lower. The aortic valve areas at baseline, 30 days, and 2 years were 1.49 ± 0.51, 1.59 ± 0.42, and 1.44 ± 0.44 cm^2^, respectively (Figure 3B). The mean aortic gradient at 30 days improved from baseline (9.9 ± 3.2 vs. 15.7 ± 11.0 mmHg, *p* < 0.01; Figure 3C). A tendency of mean aortic gradient reduction from baseline was observed at 2 years, although it was not significant (10.7 ± 3.7 vs. 15.7 ± 11.0 mmHg, *p* = 0.067). Figure 4 shows the PPM at 30 days after TAVR-in-TAVR. Eight (26%) and six (19%) patients had moderate and severe PPM, respectively. No significant differences in the creatinine concentrations were observed at 30 days compared with the baseline.

**Figure 5:**
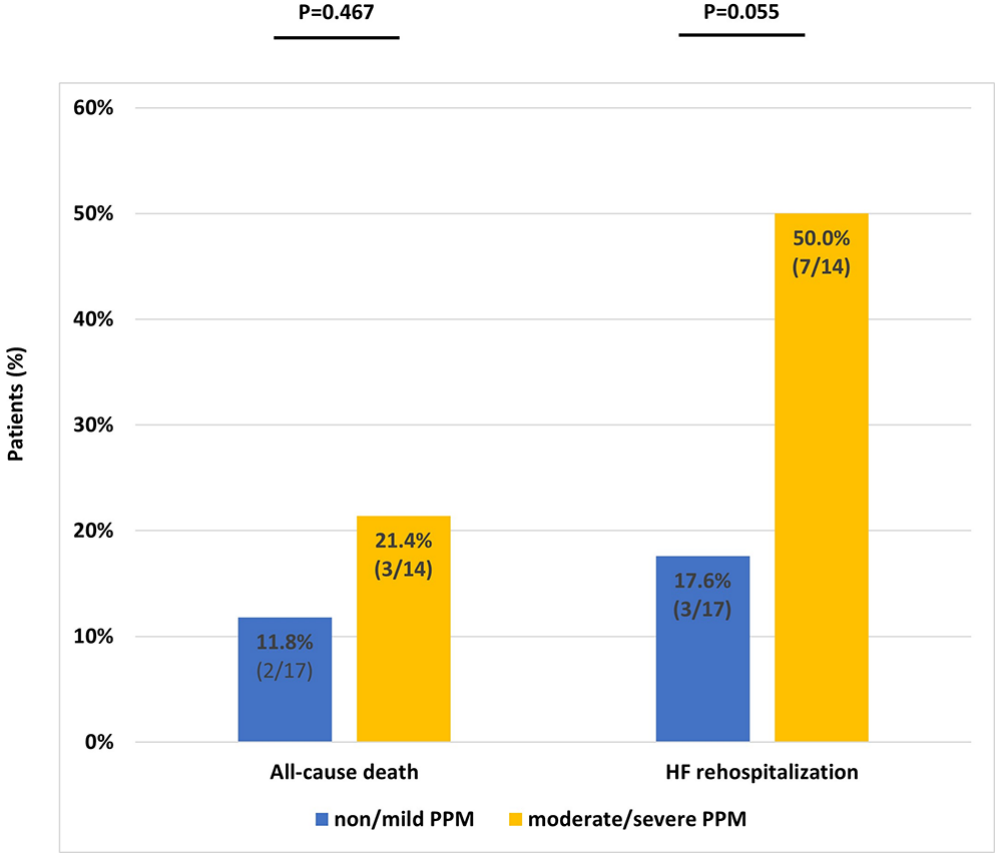
Event rates at 2 years according to prosthesis–patient mismatch severity. PPM, prosthesis–patient mismatch.

Additionally, we compared the all-cause mortality and HF-related hospitalization rates between patients with no/mild PPM and those with moderate/severe PPM. No significant differences were observed in the all-cause mortality or HF rehospitalization rates between the two groups (Figure 5). However, a trend toward higher HF readmission rates was observed in patients with moderate/severe PPM. There was a significant reduction in the B-type natriuretic peptide concentrations at 30 days from the baseline (571.8 ± 547.1 vs. 834.4 ± 957.9, *p* = 0.026; Supplementary Table S2). However, no significant difference was observed between the values at 2 years and the baseline (591.9 ± 452.6 vs. 834.4 ± 957.9, *p* = 0.075; Supplementary Figure S1).

## 4 Discussion

In recent years, TAVR-in-TAVR has emerged as a minimally invasive alternative to SAVR for the treatment of structural valve degeneration. However, TAVR-in-TAVR for PVL remains challenging due to the limited clinical experience attributable to the limited cases in clinical practice and different approaches. Additionally, there is limited research on its clinical outcomes. To the best of our knowledge, this is the first study to demonstrate the clinical outcomes of TAVR-in-TAVR for the treatment of PVL after TAVR. This study highlights the excellent success rate of TAVR-in-TAVR for PVL and accompanying reduction in severe complication rates. The long-term outcomes, including clinical functional status, were investigated, and the incidence of all-cause mortality at 2 years was acceptable. TAVR-in-TAVR also contributed to an improvement in the NYHA class. However, a high rate of HF-related rehospitalization was observed throughout the 2 years of follow-up. Echocardiographic findings showed that the prosthetic valve function was preserved after TAVR-in-TAVR during follow-up, providing encouraging insights into the durability and functionality of the procedure.

Our study cohort included high-risk older patients with high Society of Thoracic Surgery scores; however, their in-hospital complication rate was low. This suggests that TAVR-in-TAVR is also safe for high-risk patients. Furthermore, the length of hospital stay was short, which helped prevent a potential decline in the quality of life. Cerebral embolic protection devices were used in five patients (16.1%) during TAVR-in-TAVR, and they may have contributed to stroke prevention. Acute coronary obstruction is a major risk factor for valve-in-valve TAVR because coronary flow may be disturbed by the additional stent frames of the second THV (35). However, no cases of acute coronary obstruction were observed in the present study, despite the lack of techniques to prevent iatrogenic coronary artery obstruction, such as the chimney or BASILICA techniques.

The indications for TAVR-in-TAVR were considered and selected based on CT images and echocardiography findings before the procedure. VHC distance was calculated based on the analysis of preoperative CT images; values <4 mm represent a high risk of coronary artery obstruction (33, 36). The mean VTC distance for both the left and right coronary arteries was >4 mm in our study population. Hence, pre-procedural CT measurements may affect patient selection for TAVR-in-TAVR to prevent coronary artery obstruction. One of the major strengths of this study was the evaluation of preoperative CT measurements, as it is essential to prevent iatrogenic coronary artery obstruction when performing TAVR-in-TAVR.

For one patient, implantation of a larger TAVR valve (from 20 mm to 23 mm) in a smaller valve corrected a serious prosthesis-patient mismatch, addressed paravalvular leak, and demonstrated the adaptability and safety of this procedure. This decision was based on detailed measurements obtained from echo and CT imaging, underscoring the importance of thorough preoperative evaluation and personalized patient care. Nevertheless, careful patient selection is necessary because overloading with excessive TAVR can lead to serious complications such as root rupture.

Regarding the long-term outcomes, the all-cause mortality rate for TAVR-in-TAVR at 2 years was favorable, compared with the rates previously reported for patients with percutaneous PVL closure (21, 37). Previous studies have reported that newer-generation transcatheter aortic valves are associated with lower rates of mortality and complications (8, 38). In our study, newer-generation balloon-expandable TAVR valves were used in all patients for TAVR-in-TAVR. Consequently, the development of THV over time may have contributed to the favorable outcomes of TAVR-in-TAVR. Moreover, TAVR-in-TAVR markedly improved the NYHA class with a reduction in PVL. These findings revealed that TAVR-in-TAVR is beneficial in symptomatic patients with PVL. However, the rate of hospitalization for HF at follow-up in our study was relatively high. B-type natriuretic peptide concentrations improved significantly at 30 days from the baseline; however, there was no significant difference from baseline to 2 years. Some patients had moderate or worse coexisting mitral regurgitation (35.5%) or tricuspid regurgitation (22.6%), and 17 patients (54.8%) had concomitant coronary artery disease. These comorbidities may be associated with the high rate of HF-related rehospitalization.

Echocardiography confirmed that the THVs maintained their functionality and durability for up to 2 years. However, severe PPM is a common concern after TAVR, especially TAVR-in-TAVR, and it is associated with increased rates of mortality and cardiac events (39–42). A previous study from the Transcatheter Valve Therapy Registry demonstrated that severe PPM was found in 27.0% of patients undergoing valve-in-valve TAVR (43). Testa et al. reported that severe PPM after TAVR-in-TAVR was associated with increased mortality and reduced functional capacity (44). Similarly, our study confirmed a high prevalence of severe PPM in 6 patients (19%) who underwent TAVR-in-TAVR. We also found a trend toward a higher rate of HF-related rehospitalization in patients with moderate or severe PPM. These findings suggest that PPM has a negative impact on clinical outcomes of TAVR-in-TAVR for PVL. Further research is needed to explore the relationship between PPM severity and clinical outcomes in greater detail.

Understanding the mechanism underlying PVL before TAVR-in-TAVR is crucial for its effective treatment. Three main mechanisms underlie PVL after TAVR: incomplete apposition of the prosthetic valve to the annulus, an undersized prosthetic valve, and suboptimal positioning of the device. The most common cause of PVL is incomplete apposition of the prosthetic valve to the annulus due to calcified nodules or an eccentric annulus. Additional balloon dilatation may be useful for improving PVL due to an undersized prosthetic valve. Percutaneous PVL closure is viable for cases of incomplete apposition, as it helps to fill the gap between the cardiac tissue and the prosthetic valve. Saia et al. reported the outcomes of percutaneous PVL closure after TAVR and the mechanism of PVL in eligible patients, wherein incomplete attachment to the annulus was estimated to be the main cause of PVL, as observed in approximately 50% of patients (37). However, TAVR-in-TAVR is not suitable for improving PVL because of incomplete apposition to the annulus, as it is difficult to close the PVL space with a second THV implant. TAVR-in-TAVR is suitable for treating PVL due to an undersized prosthetic valve or suboptimal device positioning. Interestingly, in our study, we found that undersized prosthetic valves were the most frequent cause of PVL in 14 patients (45.2%), and suboptimal positioning, including high and low implantations, was observed in 11 patients (35.5%). Incomplete apposition of the prosthetic valve was observed in only four (12.9%) patients. Of these, two underwent concomitant percutaneous PVL closure with a vascular plug. Therefore, differences in PVL mechanisms were observed between the patients who underwent TAVR-in-TAVR and those who underwent percutaneous PVL closure. These results imply that TAVR-in-TAVR alone was not suitable for treating PVL due to incomplete apposition of the prosthetic valve. On the other hand, in the chronic phase, PVL may worsen over time. In such instances, due to factors such as valve deterioration and progressive calcification, BAV or PVL closure alone may not suffice. Performing TAVR-in- TAVR can provide higher radial force and can also overcome more of the anatomic challenges by carefully selecting the appropriate valve size and implantation depth. The PVL mechanism is important in determining the treatment strategy and device selection. Considering the mechanisms of PVL, TAVR-in-TAVR can be a reasonable procedure to reduce PVL. Our results provide valuable insights into the effectiveness and safety of TAVR-in-TAVR in patients with PVL following TAVR to improve our understanding of the potential benefits.

This study had some limitations. First, this was a retrospective observational study. Patients with PVL were carefully selected for TAVR-in-TAVR by the cardiac team, considering the anatomic features of the aortic valve or mechanisms of PVL. Selection bias could not be ruled out, given that TAVR-in-TAVR is not suitable for all patients with PVL, which may have led to skewed results. Second, only balloon-expandable valves were used for TAVR-in-TAVR. It remains unclear whether the safety and efficacy findings from our study, which exclusively used balloon-expandable valves for TAVR-in-TAVR, can be generalized to cases using self-expandable valves. Third, other concomitant procedures, such as cerebral embolic protection, percutaneous PVL closure, or balloon dilatation, were attempted in some cases. These concomitant procedures may have influenced clinical outcomes. Fourth, the sample size at the single center was relatively small, which restricts the generalizability of our findings. Future research involving a larger number of patients across multiple centers, possibly with a more diverse patient population, and longer-term outcomes is necessary to better ascertain the safety and efficacy of TAVR-in-TAVR in patients with PVL.

In conclusion, our study findings suggest that TAVR-in-TAVR is promising for selected patients with PVL following TAVR. We observed a low incidence of in-hospital complications and significant improvement in the NYHA class. Moreover, our 2-year follow-up results demonstrated a low mortality rate and favorable prosthetic valve durability, supporting the long-term safety and efficacy of TAVR-in-TAVR. However, larger, prospective studies are needed to confirm these findings and determine the optimal patient selection criteria for TAVR-in-TAVR.

## Data Availability

The original contributions presented in the study are included in the article/**Supplementary Material**, further inquiries can be directed to the corresponding author.
