# Supplementary material for: TAVR-in-TAVR with a balloon-expandable valve for paravalvular leak
Source: Front Cardiovasc Med. 2024 Mar 19;11:1374078. doi: 10.3389/fcvm.2024.1374078 (PMC10985156; doi:10.3389/fcvm.2024.1374078)
Supplement: Supplementary file 1 [file Datasheet1.pdf]

Supplementary Material

Transcatheter aortic valve replacement-in-Transcatheter aortic valve replacement with a balloon-expandable valve for paravalvular leak

Supplementary Figure 1. Change in B-type natriuretic peptide concentration over time.

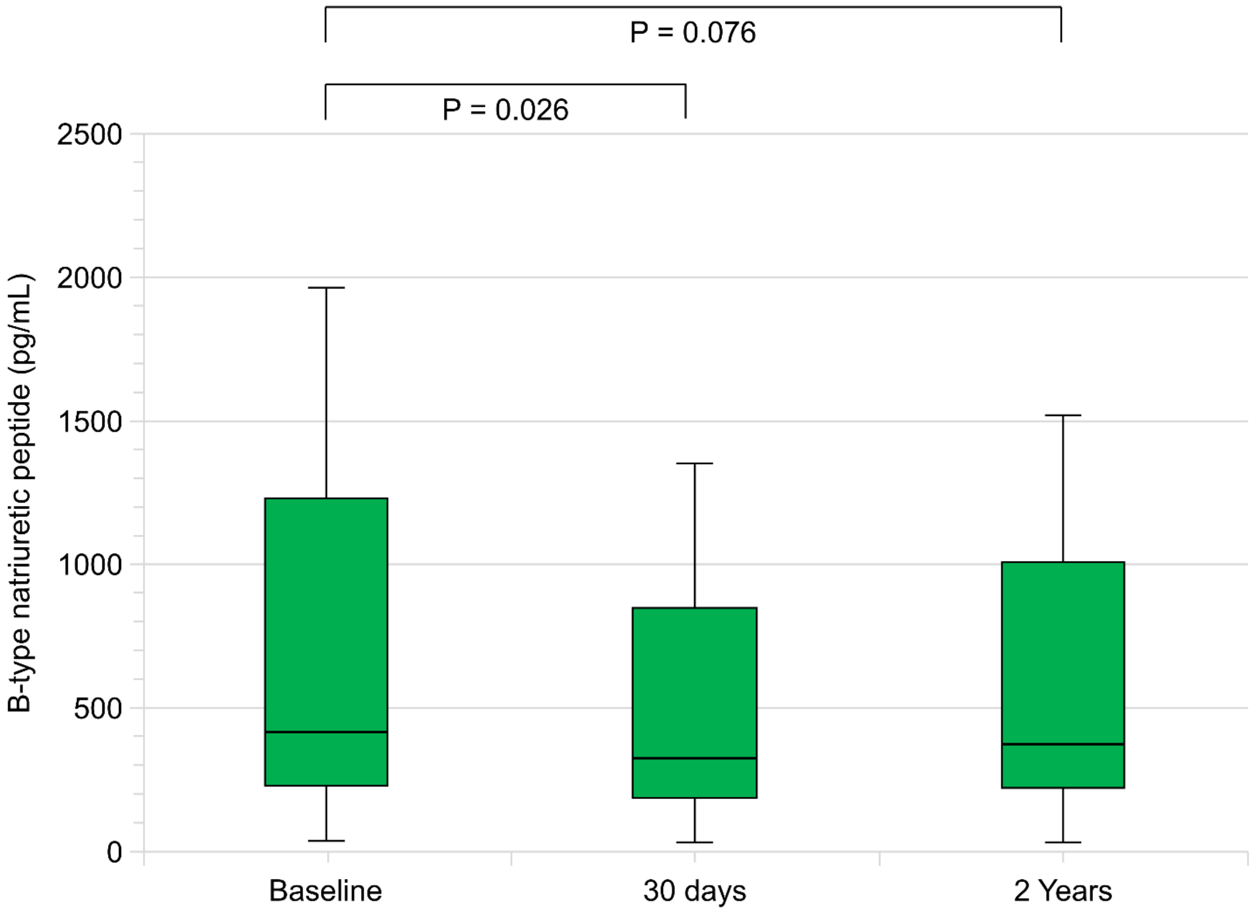

Supplementary Table 1. Procedural data of first TAVR

Patient	Prior TAVR Device	Prior THV size, mm	Access site	Urgent	Bicuspid	Cerebral embolic protection	Fluoroscopy time, min	Contrast volume, ml	Pre-dilatation Balloon	post-dilatation balloon	Length of in-hospital stay, days
1	Sapien 3	26	FA	No	No	Yes	14.9	100	No	No	2
2	Sapien 3	29	FA	Yes	No	No	12.7	40	No	No	3
3	Sapien 3	23	FA	No	No	No	15.8	60	No	No	1
4	Sapien Ultra	26	FA	No	No	No	18	60	Yes	No	4
5	Core valve	29	FA	No	Yes	No	11.5	50	No	No	2
6	Sapien 3	29	FA	No	No	No	6.58	50	No	No	3
7	Sapien 3	23	FA	Yes	No	No	14.1	70	No	No	5
8	Sapien 3	23	FA	No	No	No	8.6	25	No	No	1
9	Jenavalve	27	FA	No	No	No	unknown	unknown	No	Yes	5
10	Sapien XT	26	FA	No	No	No	17.5	120	No	No	3
11	Sapien	23	FA	No	No	No	3.2	50	Yes	No	3
12	Sapien 3	29	FA	No	Yes	No	63.1	120	No	Yes	1
13	Sapien	26	FA	No	No	No	28	150	Yes	No	3
14	Sapien	23	FA	Yes	No	No	11.5	40	Yes	No	7
15	Evolte R	26	FA	Yes	No	No	50	270	No	No	7
16	Core Valve	31	FA	No	No	No	16.4	100	Yes	No	2
17	Evolte R	29	FA	No	No	No	18.2	125	Yes	Yes	2
18	Lotus	27	FA	No	No	No	26.2	140	Yes	No	3
19	Evolte R	29	FA	No	No	No	18.3	100	Yes	Yes	2
20	Sapien Ultra	26	FA	No	No	Yes	23	80	Yes	Yes	1
21	Core valve	29	FA	No	No	No	50.9	540	Yes	Yes	7
22	Core valve	31	FA	No	No	No	unknown	unknown	No	No	4
23	Sapien 3	26	FA	No	No	Yes	38.4	290	Yes	No	1
24	Lotus	25	FA	Yes	No	Yes	27.4	100	No	No	4
25	Core Valve	29	FA	No	No	No	20.1	110	No	Yes	5
26	Sapien Ultra	26	FA	No	No	Yes	16.5	70	No	No	3

27	Sapien 3	26	FA	No	No	No	unknown	unknown	No	No	2
28	Evolut Pro	34	FA	No	No	No	15.6	70	No	No	1
29	unknown	unknown	FA	No	No	No	unknown	unknown	No	No	4
30	Core valve	31	SCA	No	No	No	unknown	unknown	No	No	4
31	Sapien 3	23	FA	No	No	No	15.8	60	No	No	3

TAVR = transcatheter aortic valve replacement; THV = transcatheter heart valve; FA = femoral artery; SCA = subclavian artery.

Supplementary Table 2. Echocardiographic and laboratory outcomes at 30 days

Echocardiography finding	At baseline	30 days post TAVR-in-TAVR	p-value
Left ventricular ejection fraction, %	54.3 ± 13.7	51.3 ± 15.7	0.38
PVL grade			<0.01
0	0 (0)	13 (41.9)	
1	0 (0)	10(32.3)	
2	4(12.9)	7(22.6)	
3	17(54.8)	0 (0)	
4	10(32.3)	1 (3.2)	
Effective orifice area, cm^2	1.49 ± 0.51	1.59 ± 0.4	0.26
Mean aortic valve gradient, mm Hg	15.7 ± 11.0	9.9 ± 3.2	<0.01
Pulmonary arterial systolic pressure, mm Hg	39.4 ± 18.9	35.7 ± 14.8	0.11
Laboratory data			
Creatinine, mg/dl	1.39 ± 0.84	1.40 ± 0.75	0.92
B-type natriuretic peptide, pg/mL	834.4 ± 957.9	571.8 ± 547.1	0.026

Values are presented as n (%) or mean ± SD.
